# Association of Preadmission Metformin Use and Prognosis in Patients With Sepsis and Diabetes Mellitus: A Systematic Review and Meta-Analysis

**DOI:** 10.3389/fendo.2021.811776

**Published:** 2021-12-23

**Authors:** Yuanzhe Li, Huayan Zhao, Yalin Guo, Yongtao Duan, Yanjun Guo, Xianfei Ding

**Affiliations:** ^1^ Department of Pediatrics, Children’s Hospital Affiliated of Zhengzhou University, Zhengzhou, China; ^2^ Department of Critical Care Medicine, The First Affiliated Hospital of Zhengzhou University, Zhengzhou, China; ^3^ General Intensive Care Unit, The First Affiliated Hospital of Zhengzhou University, Zhengzhou, China

**Keywords:** metformin, sepsis, mortality, systematic review, meta-analysis

## Abstract

**Background and Aim:**

A growing body of evidence suggests that preadmission metformin use could decrease the mortality of septic patients with diabetes mellitus (DM); however, the findings remain controversial. Therefore, this meta-analysis was conducted on available studies to confirm the relationship between preadmission metformin use and mortality in patients with sepsis and DM.

**Methods:**

A comprehensive search of the PubMed, Embase, and Cochrane Library databases was performed for studies published before August 8, 2021. Observational studies assessing the correlation between metformin use and mortality in patients with sepsis and DM were considered eligible studies. We used the Newcastle–Ottawa Scale (NOS) to assess the outcome quality of each included article. Furthermore, the odds ratios (ORs) and 95% confidence intervals (*CI*s) were analyzed using the inverse variance method with random effects modeling.

**Results:**

Eleven articles including 8195 patients were analyzed in this meta-analysis. All the included articles were scored as low risk of bias. Our results showed that preadmission metformin use had a lower mortality rate (OR, 0.74; 95% *CI*s, 0.62–0.88, *P* < 0.01) in patients with sepsis and DM. Surprisingly, there was no statistically significant difference in the levels of serum creatinine (weighted mean difference (WMD), 0.36; 95% *CI*s, −0.03–0.75; *P* = 0.84) and lactic acid (WMD, −0.16; 95% *CI*s, −0.49–0.18; *P* = 0.07) between preadmission metformin use and non-metformin use.

**Conclusions:**

This study is the most comprehensive meta-analysis at present, which shows that preadmission metformin use may reduce mortality and not increase the levels of serum creatinine and lactic acid in adult patients with sepsis and DM. Therefore, these data suggest that the potential efficacy of metformin could be assessed in future clinical studies.

**Systematic Review Registration:**

https://inplasy.com/?s=INPLASY2021100113, identifier INPLASY2021100113.

## Introduction

Sepsis is a life-threatening systemic inflammation characterized by host immune dysfunction and multiple organ damage (1). An estimated 30 million sepsis cases and 5.3 million deaths occur globally every year, indicating that sepsis is an urgent medical and health problem (2). However, the exact mechanism of sepsis development is unclear. Inflammatory factors and mediators such as high mobility group protein and nuclear factor-kappa B (NF-κB) may play significant roles in the pathogenesis of sepsis (3, 4). Recently, studies demonstrated that continual and active inflammatory molecular responses need sufficient metabolic supply. Thus, adjustments targeting metabolic pathways are a novel potential therapeutic strategy (5). Metformin reduces the inflammatory response in the body, which may reduce mortality in septic patients with diabetes mellitus (DM).

Metformin, a classic oral antidiabetes drug, is extensively recommended as a first-line treatment of type 2 DM (6, 7). In addition to the well-known hypoglycemic effects of metformin, evidence shows that metformin plays an anti-inflammatory role by inhibiting the expression of inflammatory factors (8–12). Metformin may also inhibit the respiratory chain complex I of the electron transport chain to increase the adenosine monophosphate/adenosine triphosphate ratio, and consequently, induce adenosine 5′-monophosphate-activated protein kinase (AMPK) activation. AMPK interferes with inflammation and other molecular processes. Given the AMPK activation, metformin may be a potential therapeutic therapy for sepsis (13).

Recent studies demonstrated the positive effects of metformin in patients with sepsis. Doenyas-Barak et al. (14) and Jochmans et al. (15) suggested that pre-exposure of patients to metformin lowered mortality compared with control, although metformin was associated with higher lactate concentrations. Green et al. (16) reported that patients not taking metformin had 2.5 times more risk of dying within 28 days than those taking metformin. However, no statistical differences in mortality between metformin users and nonusers were detected in acute respiratory distress syndrome, DM, sepsis, and critically ill patients (17–19). As the effects of metformin on mortality are controversial, we investigated the effects of metformin on mortality in patients with sepsis and DM.

## Methods

This systematic review and meta-analysis protocol was registered on INPLASY (ID: INPLASY2021100113). This study was conducted according to the guidance of the meta-analysis of observational studies in the epidemiological guidelines. The PRISMA 2020 checklist is shown as **Supplemental Table 1**.

### Search Strategy

We comprehensively searched the PubMed, Cochrane, and Embase databases for English language studies published before August 08, 2021. We used a combination of MeSH/Emtree, title, abstract, and keyword terms to search for relevant studies. The search terms were “metformin”, “sepsis”, and “critically ill”. Endnote x9 software was used for literature screening. We reviewed the eligible articles to identify other potentially relevant studies. Literature retrieval was conducted by two researchers. The search process is shown in **Supplemental Table 2**.

### Inclusion Criteria

Studies were included in this meta-analysis if: the patients with sepsis and DM who used metformin before admission were enrolled; the control group was DM complicated with sepsis but not treated with metformin; mortality in metformin and non-metformin users was measured; the patients were adults; the articles were observational studies; and the articles were published in English. Articles lacking relevant outcomes or patients with sepsis complicated with other illnesses were excluded. Commentaries, reviews, and studies for which full articles could not be retrieved were excluded.

### Eligible Studies and Extracted Data

The relevant articles and eligible data were assessed and extracted by two authors, respectively. If a disagreement occurred, which was discussed and the consensus with a third author was reached. The following data were collected from each study: first author name, publication date, the type of studied design and center, number of patients, study period, sex and mean age between groups and primary outcome.

### Risk of Bias Evaluation

Using the Newcastle–Ottawa Scale (NOS) for cohort studies, the risk of bias was assessed for each outcome in all included studies. According to the selection of cohort (up to 4 points), the comparability of cohort design and analysis (up to 2 points) and the adequacy of result measurement (up to 3 points), a maximum of 9 points will be obtained. Seven to nine points are considered high quality (low risk of bias) (20).

### Statistical Analysis

The interesting outcomes were mortality in sepsis patients with or without metformin before admission. Furthermore, the meta-analysis used the combined effects of each result. We calculated odds ratios (ORs) and 95% confidence intervals (*CI*s) for each result using a random effects model to explore the heterogeneity between studies.

The outcome of interest was the mortality, serum creatinine and lactic acid levels of septic patients with or without preadmission metformin use. The meta-analysis used the pooled effects of each outcome. The random effects models were used to evaluate ORs and 95% *CI*s of each outcome to investigate the heterogeneity between studies. Furthermore, we used the *I* ^2^ and *P* values to assess the heterogeneity. When *I* ^2^ was 51%–74%, the percentage of variation caused by heterogeneity rather than sampling error was considered medium, while it was high when *I* ^2^ was ≥ 75%. Begg’s funnel plot (21) was used to assess possible publication bias. We visually assessed the asymmetry of the funnel plots. For Begg’s funnel, *P* < 0.1 suggested that the scale of the study is very small. All statistical analyses were performed using Stata 14.0 (College Station, Texas, 77845, USA).

## Results

### Study Selection

**Figure 1:**
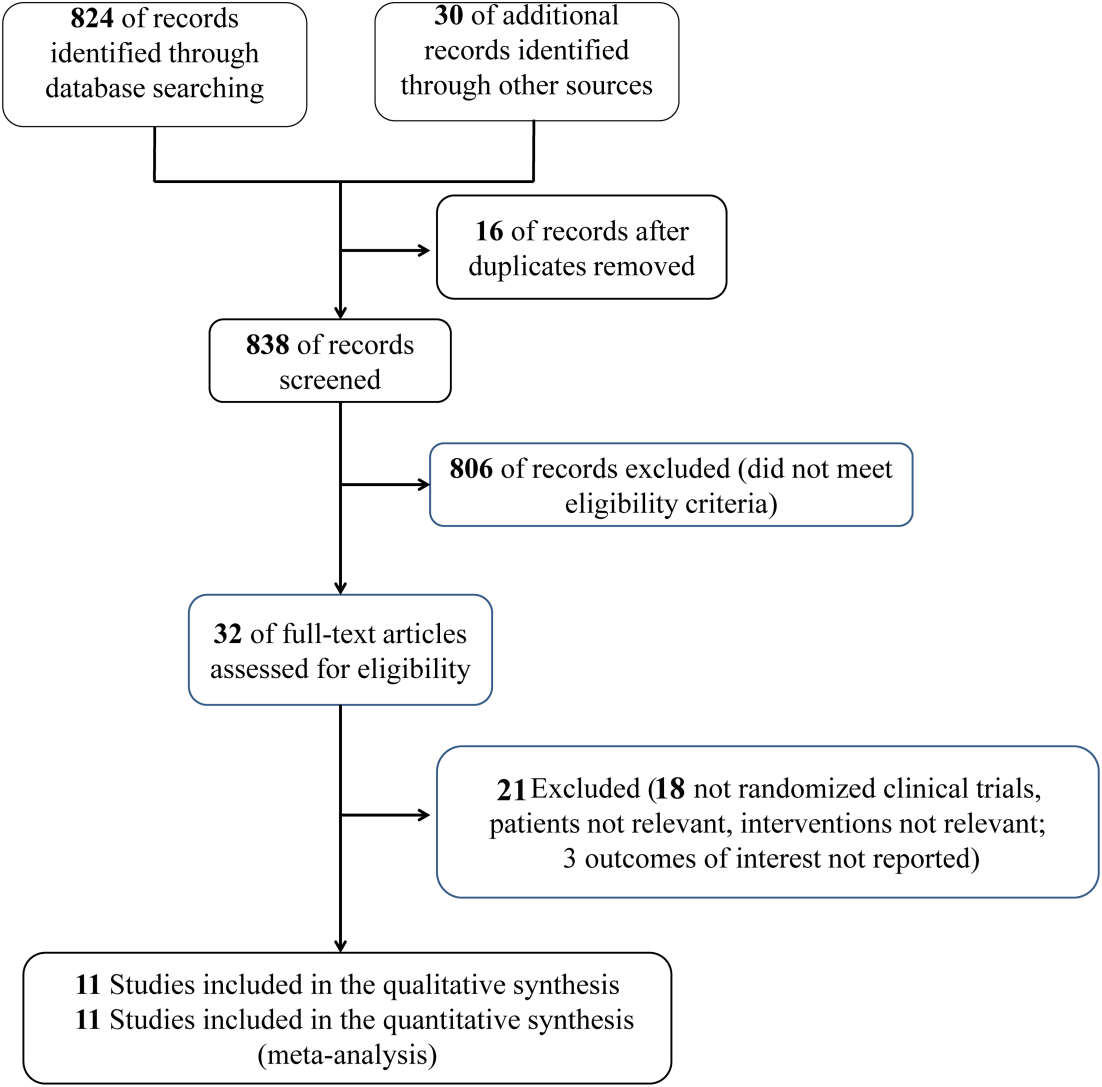
Flowchart of the study selection process.

854 studies were determined and 16 studies were eliminated because of duplication. After assessing the full-text eligibility, only 32 articles that addressed reasons potentially associated with the original study question remained. Eleven studies (14–19, 21–25) enrolling 8195 patients were included in the meta-analysis. **Figure 1** shows the process of study selection.

### Study Characteristics

The eligible studies were observational studies that included septic patients with DM who used preadmission metformin. Furthermore, three articles (16, 17, 21) showed 28 day mortality, four studies (18, 19, 22, 23) reported 30 day mortality, and four studies (14, 15, 24, 25) reported in-hospital mortality. The primary outcome data were extracted. If ORs and 95% *CI*s were missing, the data were calculated based on the original data reported from the original studies. **Table 1** presents the baseline information regarding the analyzed studies.

**Table 1:** Characteristics of identified studies.

Study	Study design	M/S Centre	No. of MET group	No. of NM group	Female/Male in MET group	Female/Male in NM group	Mean age in MET group	Mean age in NM group	Study period	Primary Outcome
(24)	RC	Single	12	53	NA	NA	NA	NA	12/2005-6/2009	Incidence of LA
(16)	RC	Single	192	343	NA	NA	71	72	02/2007-10/2008	28d mortality
(25)	RC	Single	114	127	NA	NA	NA	NA	01/2011-07/2013	mortality
(23)	RC	Multi	73	182	NA	NA	NA	NA	01/2005-12/2011	30d mortality
(18)	PO	Multi	114	127	36/78	61/66	67	65.1	01/2011-07/2013	Hospital mortality
(14)	RC	Single	44	118	22/22	47/71	74	68	01/2011-06/2013	Hospital mortality
(21)	RC	Single	71	142	32/39	62/80	67	68	08/2008-09/2014	28d mortality
(15)	RC	Single	52	79	19/33	23/56	66	71	10/2010-12/2013	Hospital mortality
(17)	RC	Single	162	162	97/65	87/75	69	69	01/2007-12/2013	28d mortality
(19)	RC	Single	672	672	364/308	359/313	69.1	69.1	2011-2015	30d mortality
(22)	RC	Multi	476	1907	246/230	992/915	70.1	69.5	2001-2012	30d mortality

RC, retrospective cohort; PO, prospective observational study; M, multiple; S, single; MET, metformin; NM, non-metformin; NA, Not available; LA, lactic acidosis.

### Risk of Bias Evaluation

Ten studies were observational studies, and one was a prospective cohort study. The risk of bias assessment scores for all studies was greater than or equal to six points, indicating a low risk of bias based on the NOS. **Supplemental Table 3** shows the details of the risk of bias for the eligible studies.

### Effects of Metformin on Outcomes

**Figure 2:**
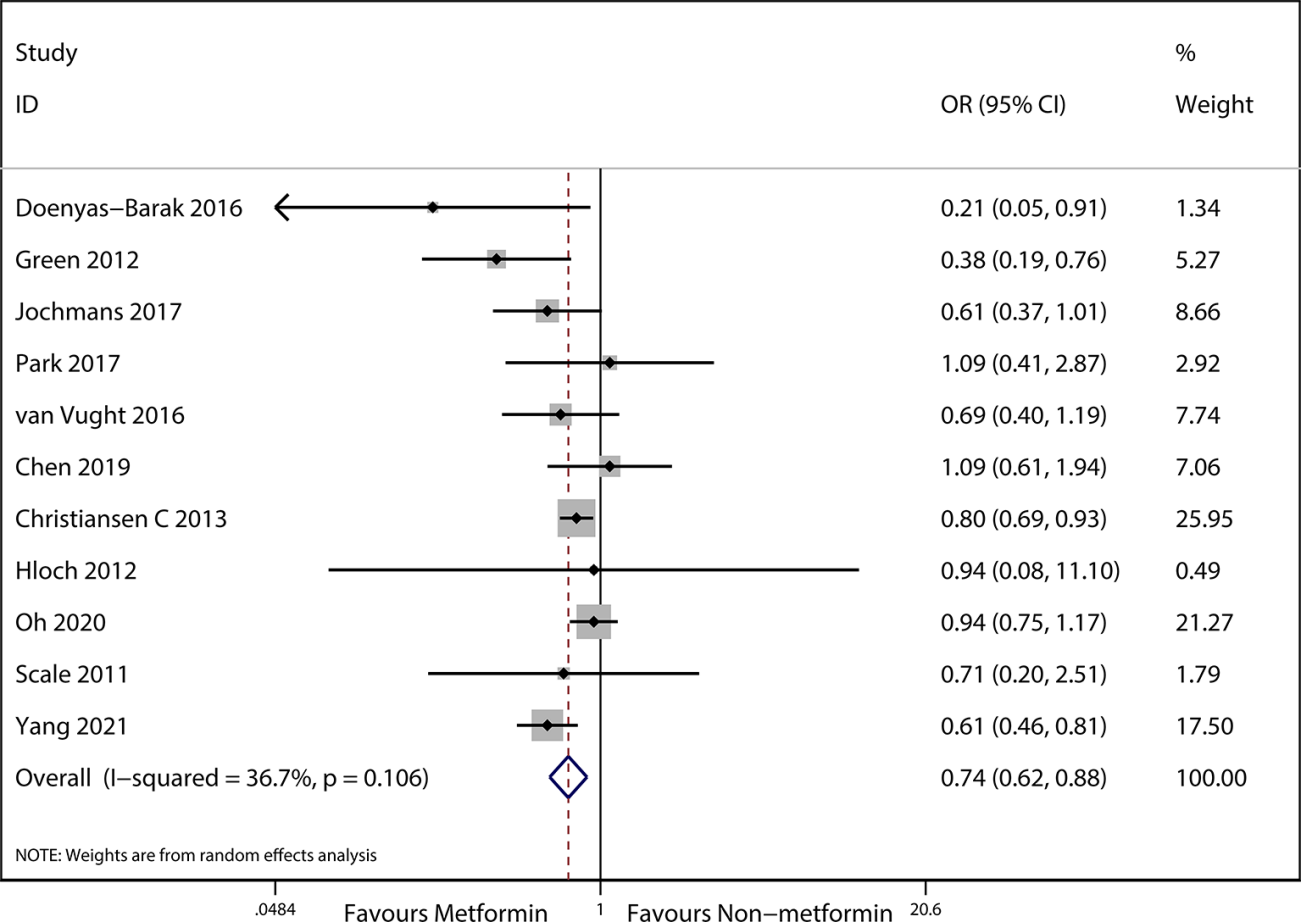
Meta-analysis of the overall pooled ORs of studies investigating the mortality outcomes of patients with sepsis and DM. The Forest plot shows the significance of the association between metformin use and mortality in patients with sepsis and DM according to the random effects model.

**Figure 3:**
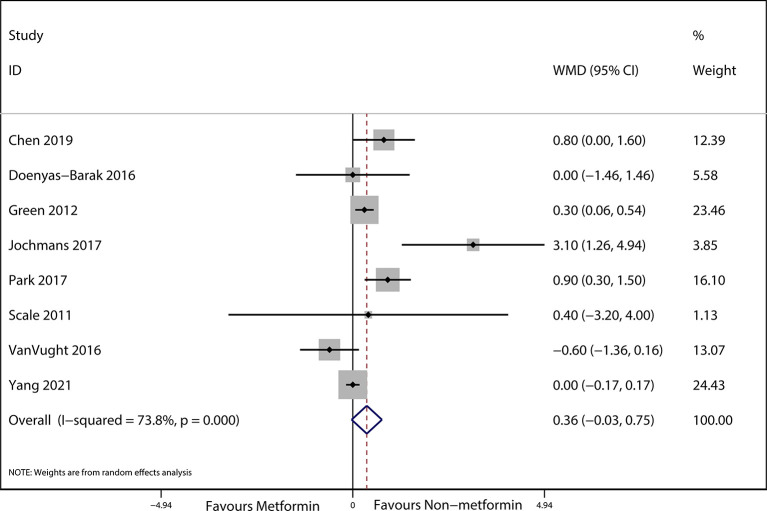
Meta-analysis of the ORs of included articles researching the serum lactic acid of septic patients with DM. The outcome of Forest plot indicated the relation between preadmission metformin use and serum lactic acid in septic patients with DM based on the random effects model.

**Figure 4:**
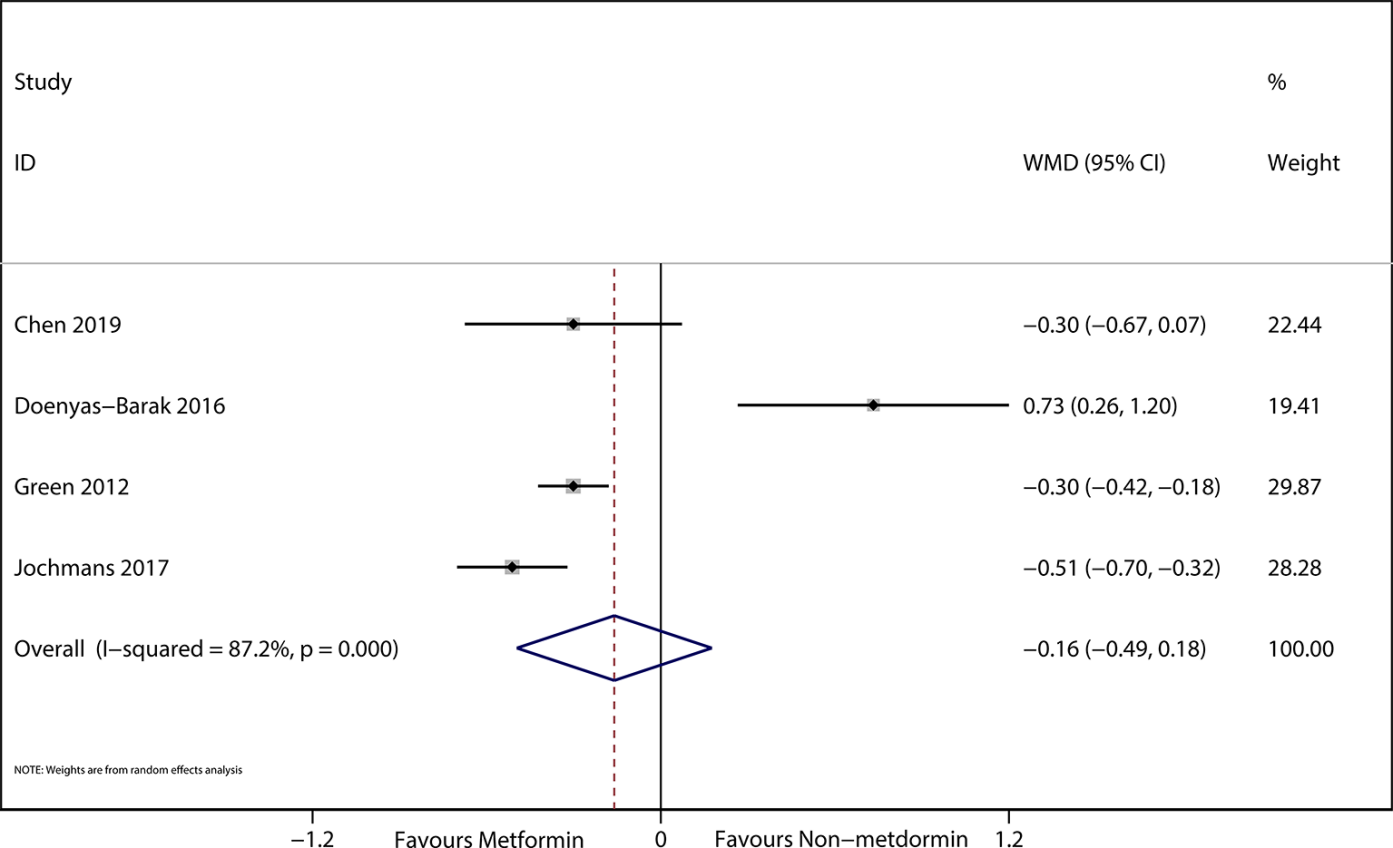
The Forest plot suggested the association between preadmission metformin use and serum creatinine in septic patients with DM.

The preadmission use of metformin in septic patients significantly lowered mortality when compared with the mortality in patients who were not treated with metformin (OR, 0.74; 95% *CI*s, 0.62–0.88; *P* < 0.01; **Figure 2**). No significant differences in serum creatinine concentrations (weighted mean difference (WMD), 0.36; 95% *CI*s, −0.03–0.75; *P* = 0.84; **Figure 3**) were detected between patients taking metformin and those not taking metformin. Similarly, no significant differences in serum lactic acid concentrations (WMD, −0.16; 95% *CI*s, −0.49–0.18; *P* = 0.07; **Figure 4**) were detected between patients taking metformin and those not taking metformin.

### Sensitivity Analyses

The eligible studies were observational articles with a low risk of bias (**Supplemental Table 3**). We conducted a sensitivity analysis to evaluate the influence of any study on the ORs and 95% *CI*s by omitting an individual study at a time. The data indicated that the results of mortality and lactic acid were robust and reliable (**Supplemental Figures 1**, **2**).

### Evaluation of Publication Bias

We performed the Funnel plots (**Supplemental Figures 3**, **4**) and Begg’s funnel plot (**Supplemental Figures 5**, **6**) to assess the publication bias in the included articles between mortality and lactic acid in this analysis. No publication bias of them was found (*P* = 0.291 and *P* = 0.291, respectively).

## Discussion

This meta-analysis included 8195 patients and demonstrated that the mortality rate of preadmission metformin users was lower than that of non-metformin users in adults with DM and sepsis. Our findings suggest that metformin could have a therapeutic potential for septic patients with DM.

The relationship between metformin use and mortality in patients with infectious diabetes remains controversial. Therefore, some studies (17–19) demonstrated no significant differences between metformin use and non-metformin use in patients with sepsis. In contrast, the reports of Green et al. (16) and Doenyas Bara et al. (14) showed that preadmission metformin use in septic patients significantly lowered mortality compared with those not using metformin. Subsequently, a meta-analysis (26) only including five observational cohort studies (1282 patients) suggested that preadmission metformin use could decrease the 28-d mortality in septic patients with DM. As the sample size of the study (26) was too small, the conclusions should be confirmed in the large sample study, especially in clinical studies. The recent large sample study (19) showed that preadmission metformin use was not significantly related to the risk of sepsis and 30-d mortality of septic patients with DM. Comparatively, the latest study (22) indicated that preadmission metformin use was associated with a 39% decrease in 30-d mortality in septic patients with DM. Therefore, the conclusions still exist in controversy among the available articles. This meta-analysis provides this comprehensive evidence that metformin use reduces mortality in septic patients with DM.

The mechanism by which metformin reduces mortality in patients with sepsis is unclear. The 2016 definition of sepsis includes lactate concentration and measurement of creatinine concentration to determine the progress of organ failure (1). Furthermore, metformin has a low propensity for hyperlactatemia (27, 28). Previous studies (29, 30) showed that mortality due to lactic acidosis caused by metformin was lower than other forms of lactic acidosis. Intriguingly, a study showed that lactic acid could be considered an energy source and can provide energy, such as glucose, amino acids, and ketones for ischemic tissues (31). This may explain the results in the previous study (32) that the plasma concentration of lactic acid in surviving patients was higher than that in dying patients. Reciprocally, in our study, creatinine and lactate concentrations were not significantly different in patients with and without metformin (14–18, 21, 22). Additionally, increasing evidence was provided to show that metformin could ameliorate the autophagy and mitochondrial function of T-cells (33) and improve the systematic inflammation *via* decreased pro-inflammatory factors, such as NF-κB and tumor necrosis factor alpha (TNF-α) (34). Moreover, metformin reduces oxidative stress, enhances antioxidant defense (35), improves insulin resistance, and protects vascular endothelium (36). Notably, metformin upregulates the AMPK activation (13, 34), which is considered a potential therapeutic agent for sepsis-associated organ injury (37). Furthermore, a recent study suggested that the AMPK-dependent immunometabolism pathway disorder may conduce to an increased risk of sepsis (38). Finally, metformin may have antibacterial effects (39), improving the prognosis of sepsis in metformin users.

Meta-analysis is a comprehensive statistical analysis method for multiple studies on a subject. When the difference between the results of each study is greater than expected, there is statistical heterogeneity in the summary results of the meta-analysis. The high heterogeneity of the included studies of this study reflects the differences of these studies. Because of the low publication bias, we consider the heterogeneity was not significant. The heterogeneity may originate from differences in sample sizes, lactate concentrations, and the combined application of other antidiabetes drugs.

This meta-analysis has several strengths. Firstly, the sample size was large, making the results more convincing. Secondly, the risk of bias was low for all included studies. Finally, the random effects model with generic inverse variance was used, and the adjusted ORs and 95% *CI*s were extracted to compute the effect of metformin on mortality.

Our study has several limitations. Despite the comprehensive search, only eleven studies met the inclusion standards and ten were observational studies; therefore, the conclusion should be confirmed in future clinical studies. Furthermore, most original studies did not report the dose of metformin and whether other kinds of hypoglycemic agents were used. Hence, this study did not determine whether different doses of preadmission metformin use and other hypoglycemic agents would influence the effect of metformin on septic patients with DM. Thirdly, this study was constrained to studies published in English. Thus, publication bias cannot be excluded.

This meta-analysis suggests that we should continue perform the large sample observational and clinical study to confirm the effect of metformin on septic patients with DM, and thereby to searche the mechanism of metformin in reducing the mortality on septi patients with DM. Additionally, the does and time of metformin use in septic patients with DM should be researched in the future clinical studies.

## Conclusion

This is the most comprehensive meta-analysis to investigate the association of metformin use with mortality in septic patients with DM. The findings suggest that preadmission metformin use may reduce the mortality of septic patients with DM, and without increasing the levels of serum creatinine and lactic acid. However, stronger support from more high-quality research is still needed.

## Author Contributions

All the authors contributed equally to the work presented in this article. YL, HZ, YjG, and XD conceived the idea of this study. YlG and YD contributed to the data extraction. YL and HZ computed and evaluated the pooled outcomes. YjG and XD contributed to the study protocol and wrote the article. YjG and XD revised the article. All authors contributed to the article and approved the submitted version.

## Funding

This study was supported by the 2021 youth talent promotion project in Henan Province (Grant No.2021HYTP053), 2021 joint construction project of Henan Medical Science and technology breakthrough plan (Grant No. LHGJ20210299), and Henan Provincial Ministry Co-construction Project (2018010040).

## Conflict of Interest

The authors declare that the research was conducted in the absence of any commercial or financial relationships that could be construed as a potential conflict of interest.

## Publisher’s Note

All claims expressed in this article are solely those of the authors and do not necessarily represent those of their affiliated organizations, or those of the publisher, the editors and the reviewers. Any product that may be evaluated in this article, or claim that may be made by its manufacturer, is not guaranteed or endorsed by the publisher.

## Supplementary Material

Supplementary Figure 1 Funnel plot assessing the mortality after preadmission metformin use in septic patients with DM.

Supplementary Figure 2 Funnel plot evaluating serum lactic acid after preadmission metformin use in patients with sepsis and DM.

Supplementary Figure 3 Sensitivity analysis indicating that the included studies were conclusive and reliable regarding preadmission metformin use and the mortality of septic patients with DM.

Supplementary Figure 4 Sensitivity analysis indicating that the included studies were conclusive and reliable regarding preadmission metformin use and the serum lactic acid of septic patients with DM.

Supplementary Figure 5 Begg’s funnel plot assessing the mortality of preadmission metformin use in patients with sepsis and DM.

Supplementary Figure 6 Begg’s funnel plot assessing the serum lactic acid of preadmission metformin use in patients with sepsis and DM.
