# Supplementary material for: Association of Preadmission Metformin Use and Prognosis in Patients With Sepsis and Diabetes Mellitus: A Systematic Review and Meta-Analysis
Source: Front Endocrinol (Lausanne). 2021 Dec 23;12:811776. doi: 10.3389/fendo.2021.811776 (PMC8735596; doi:10.3389/fendo.2021.811776)
Supplement: Supplementary Figure 1 — Funnel plot assessing the mortality after preadmission metformin use in septic patients with DM. [file DataSheet_1.zip › Data sheet 1/Supplemental Table 2.docx]

**Supplemental Table 2. The detailed search strategy**

| **Electronic databases** | **Search** | **Search strategy** | **Results** |
| --- | --- | --- | --- |
| **Pudmed** | #1 | (((sepsis[MeSH Terms]) OR (sepsis[Title/Abstract])) OR (Critically ill patients[MeSH Terms])) OR (Critically ill patients[Title/Abstract]) | 221,691 |
|  | #2 | (metformin[MeSH Terms]) OR (metformin[Title/Abstract]) | 20,458 |
|  | #3 | ((((sepsis[MeSH Terms]) OR (sepsis[Title/Abstract])) OR (Critically ill patients[MeSH Terms])) OR (Critically ill patients[Title/Abstract])) AND ((metformin[MeSH Terms]) OR (metformin[Title/Abstract])) | **146** |
| **EMBASE** | #1 | ('sepsis'/de OR sepsis:ab,ti OR 'critically ill patient':ab,ti OR 'critically ill patient'/de) | 278,977 |
|  | #2 | ('metformin'/de OR metformin:ab,ti) | 74,862 |
|  | #3 | #1 AND #2 | **646** |
| **Cochrane CENTRAL** | #1  #2  #3  #4  #5  #6  #7  #8  #9 | (sepsis):ti,ab,kw  MeSH descriptor: [Sepsis] this term only  ("critically ill"):ti,ab,kw  MeSH descriptor: [Critical Illness] this term only  #1 OR #2 OR #3 OR #4  (metformin):ti,ab,kw  MeSH descriptor: [Metformin] this term only  #6 OR #7  #5 AND #8 | 11,818  2,181  7,413  2,466  18,790  11,404  4,304  11,404  **32** |
