# Supplementary material for: Association of Preadmission Metformin Use and Prognosis in Patients With Sepsis and Diabetes Mellitus: A Systematic Review and Meta-Analysis
Source: Front Endocrinol (Lausanne). 2021 Dec 23;12:811776. doi: 10.3389/fendo.2021.811776 (PMC8735596; doi:10.3389/fendo.2021.811776)
Supplement: Supplementary Figure 1 — Funnel plot assessing the mortality after preadmission metformin use in septic patients with DM. [file DataSheet_1.zip › Data sheet 1/Supplemental Table 3.docx]

| **Studies** | **Selection** | | | **Comparability** | | | **Assessment of outcome** | | |  |  |
| --- | --- | --- | --- | --- | --- | --- | --- | --- | --- | --- | --- |
| First author | Represent-ativeness of exposure arm(s) | Selection  of the comparati-vearm(s) | Origin of exposure source | Demonstration that outcome of interest was not present at start of study | Studies controlling the most important factors | Studies controlling the other main factors | Assessment of outcome with independenc-y | Adequacy of follow-up length (to assess outcome) | Lost to follow-up acceptable (less than 10% and reported) | Total  quality  score | |
| Green et al 2012 | * | * | * | * | * | * | * | * | - | 8 | |
| Doenyas-Barak et al 2016 | * | * | * | * | * | * | * | * | - | 8 | |
| Park et al 2017 | * | * | * | * | * | * | * | * | - | 8 | |
| VanVught et al 2016 | * | * | * | * | * | - | * | * | - | 7 | |
| Jochmans et al 2017 | * | * | * | * | * | * | * | * | - | 8 | |
| Chen et al 2019 | * | * | * | * | * | * | * | * | - | 8 | |
| Oh et al 2020 | * | * | * | * | * | * | * | * | - | 8 | |
| Yang et al 2021 | * | * | * | * | * | * | * | * | - | 7 | |

**Supplemental Table 3.** The Newcastle-Ottawa quality assessment scale of including studies.

**Supplemental Table 3 Continued.**

| **Studies** | **Selection** | | | **Comparability** | | | **Assessment of outcome** | | |  |  |
| --- | --- | --- | --- | --- | --- | --- | --- | --- | --- | --- | --- |
| First author | Represent-ativeness of exposure arm(s) | Selection  of the comparati-vearm(s) | Origin of exposure source | Demonstration that outcome of interest was not present at start of study | Studies controlling the most important factors | Studies controlling the other main factors | Assessment of outcome with independenc-y | Adequacy of follow-up length (to assess outcome) | Lost to follow-up acceptable (less than 10% and reported) | Total  quality  score | |
| Christiansen et al 2013 | * | * | * | * | * | * | * | * | - | 8 | |
| Scale et al 2011 | - | * | * | - | * | * | * | * | - | 6 | |
| Hloch et al 2012 | * | * | * | * | * | - | * | * | - | 7 | |
