# Supplementary figures and images for: Association of Preadmission Metformin Use and Prognosis in Patients With Sepsis and Diabetes Mellitus: A Systematic Review and Meta-Analysis
Source: Front Endocrinol (Lausanne). 2021 Dec 23;12:811776. doi: 10.3389/fendo.2021.811776 (PMC8735596; doi:10.3389/fendo.2021.811776)

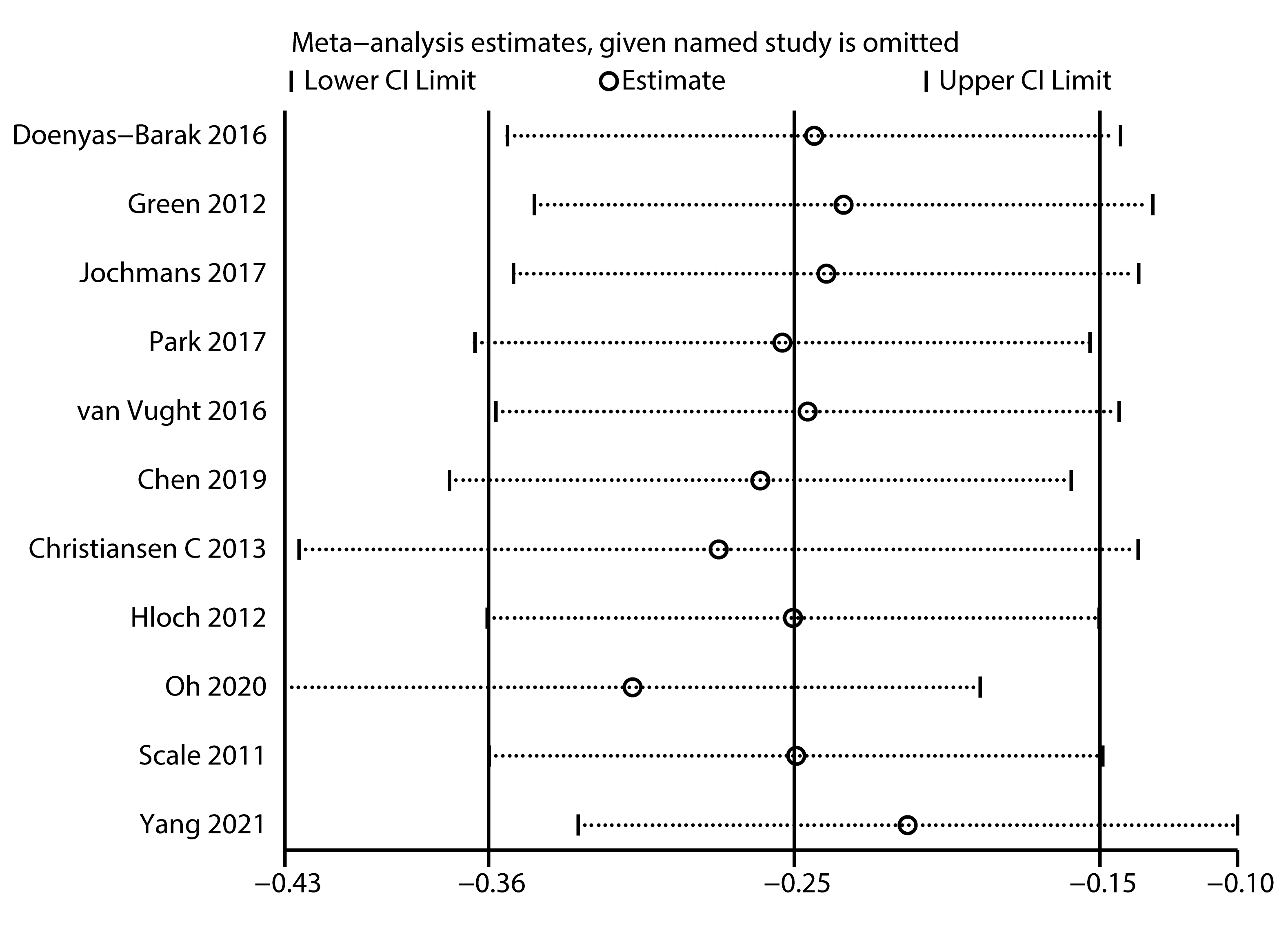

Supplement: Supplementary Figure 1 — Funnel plot assessing the mortality after preadmission metformin use in septic patients with DM. [file DataSheet_1.zip › Data sheet 1/Supplemental Figure 1.tif]

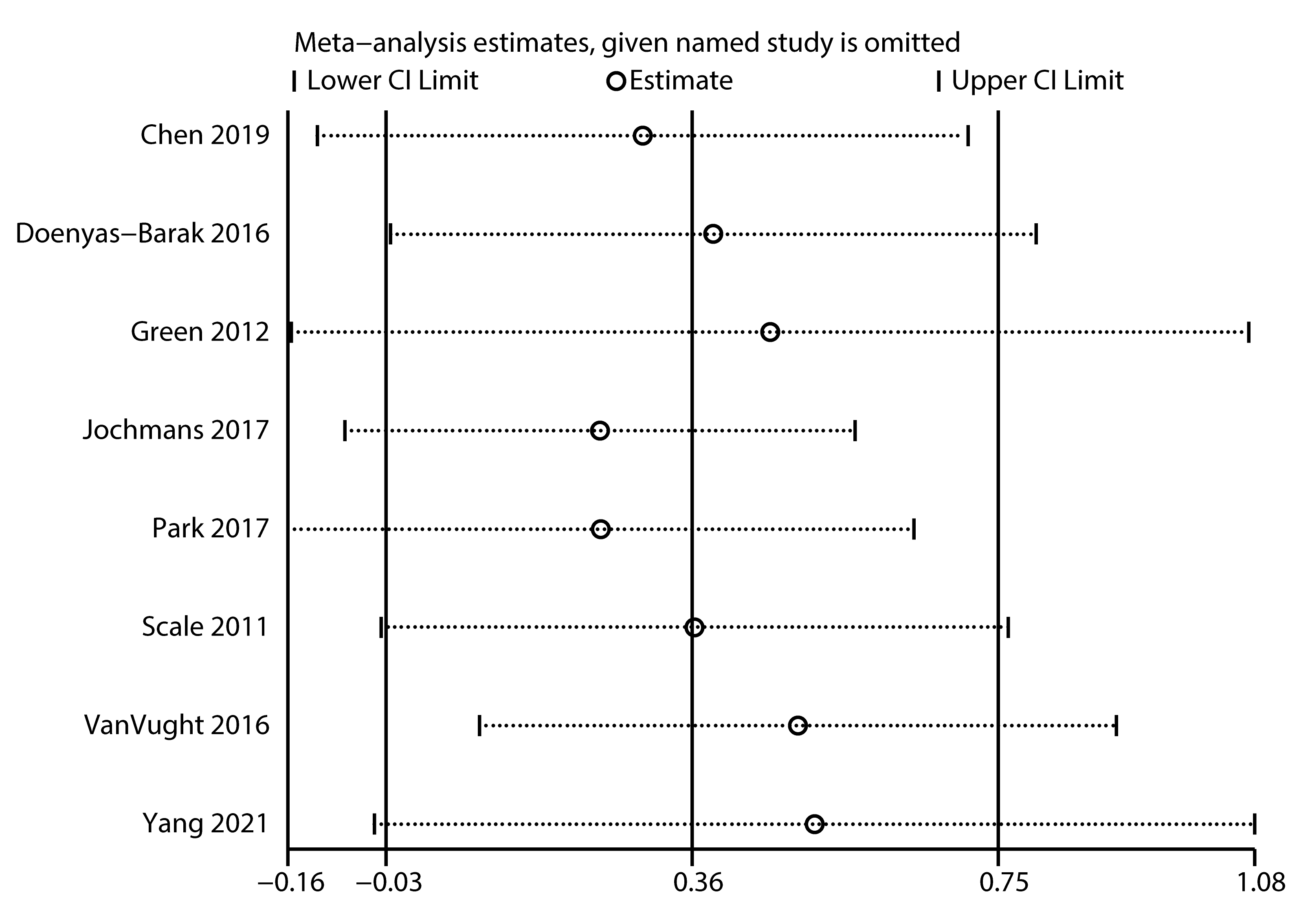

Supplement: Supplementary Figure 1 — Funnel plot assessing the mortality after preadmission metformin use in septic patients with DM. [file DataSheet_1.zip › Data sheet 1/Supplemental Figure 2.tif]

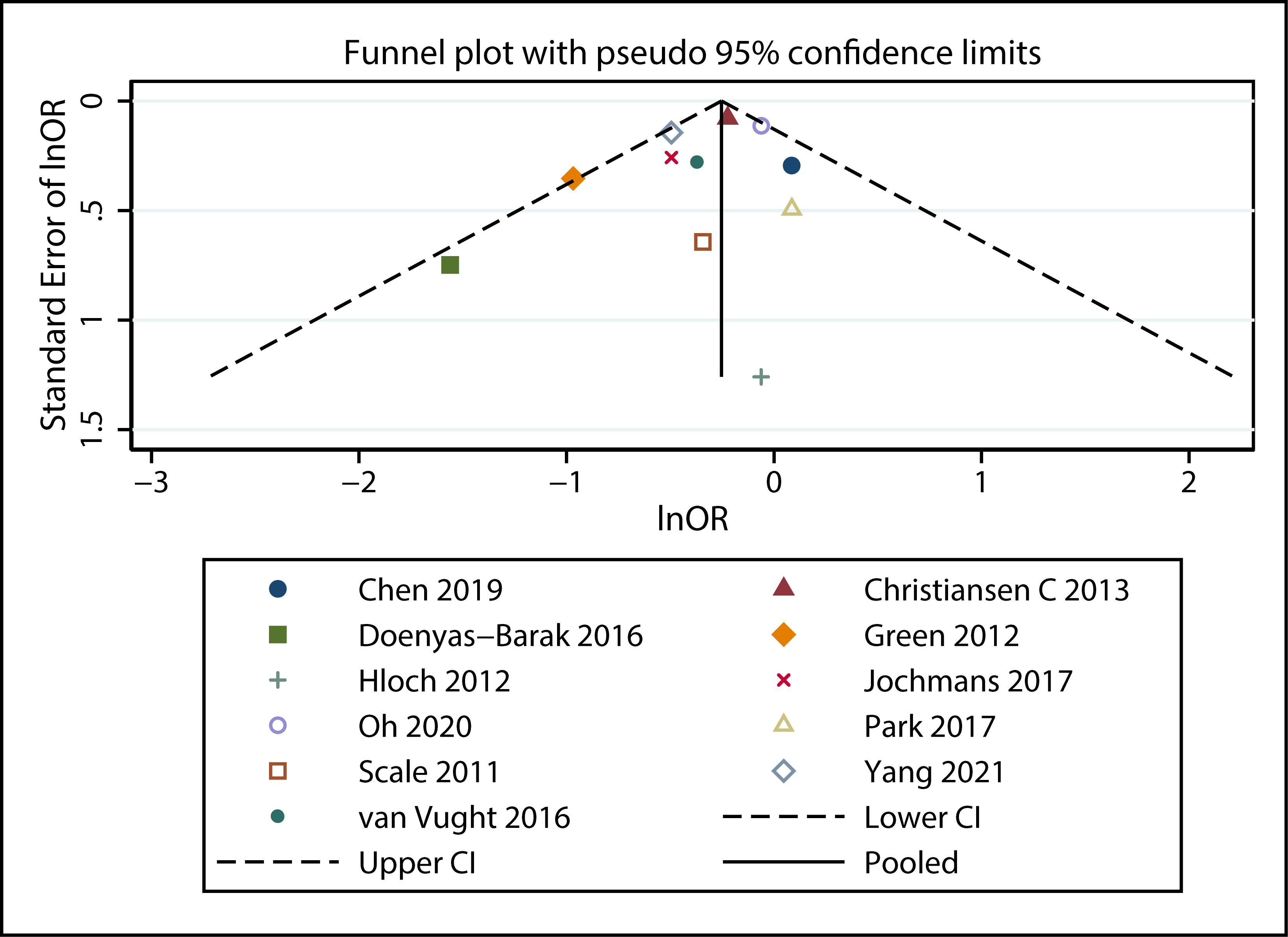

Supplement: Supplementary Figure 1 — Funnel plot assessing the mortality after preadmission metformin use in septic patients with DM. [file DataSheet_1.zip › Data sheet 1/Supplemental Figure 3.tif]

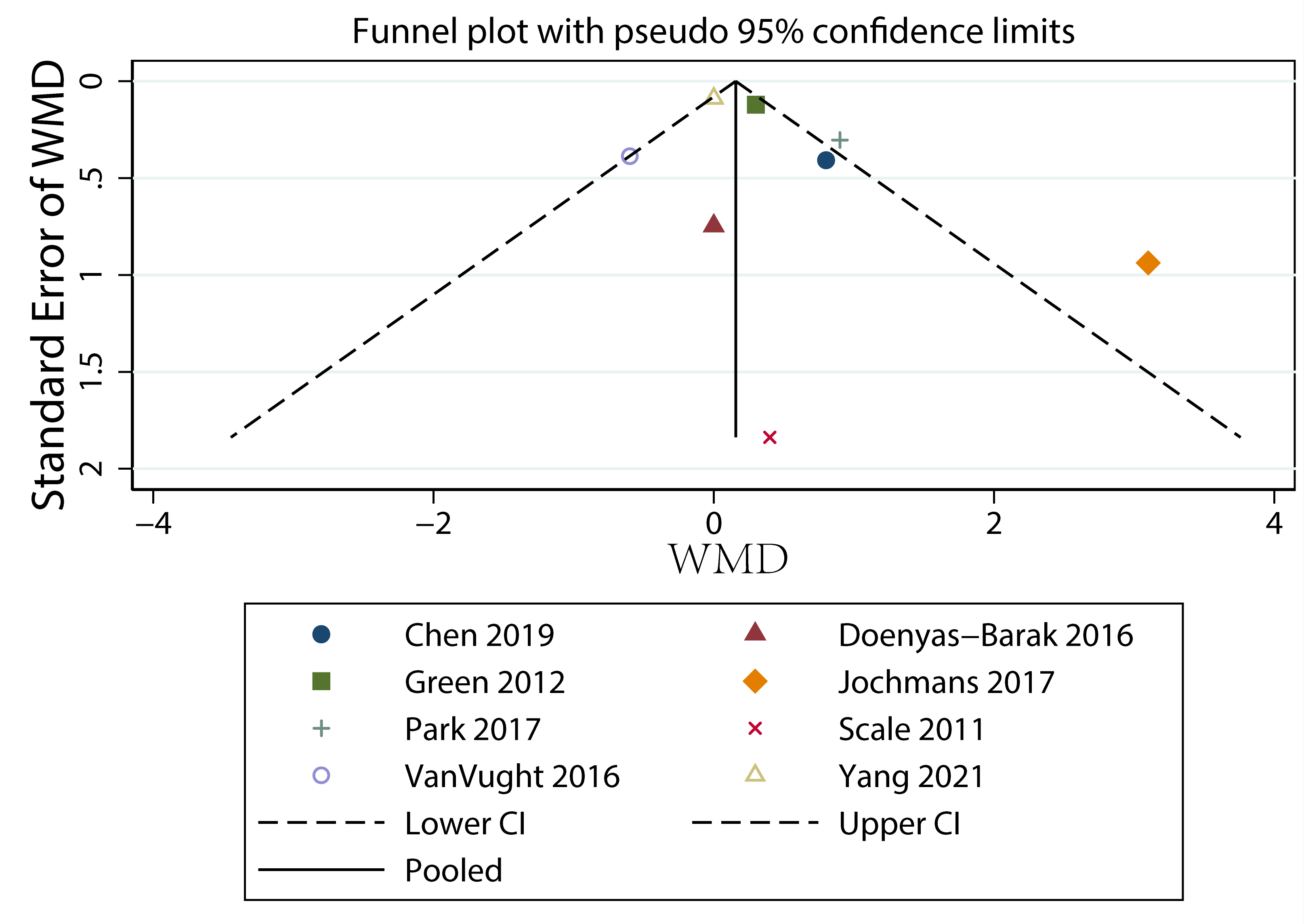

Supplement: Supplementary Figure 1 — Funnel plot assessing the mortality after preadmission metformin use in septic patients with DM. [file DataSheet_1.zip › Data sheet 1/Supplemental Figure 4.tif]

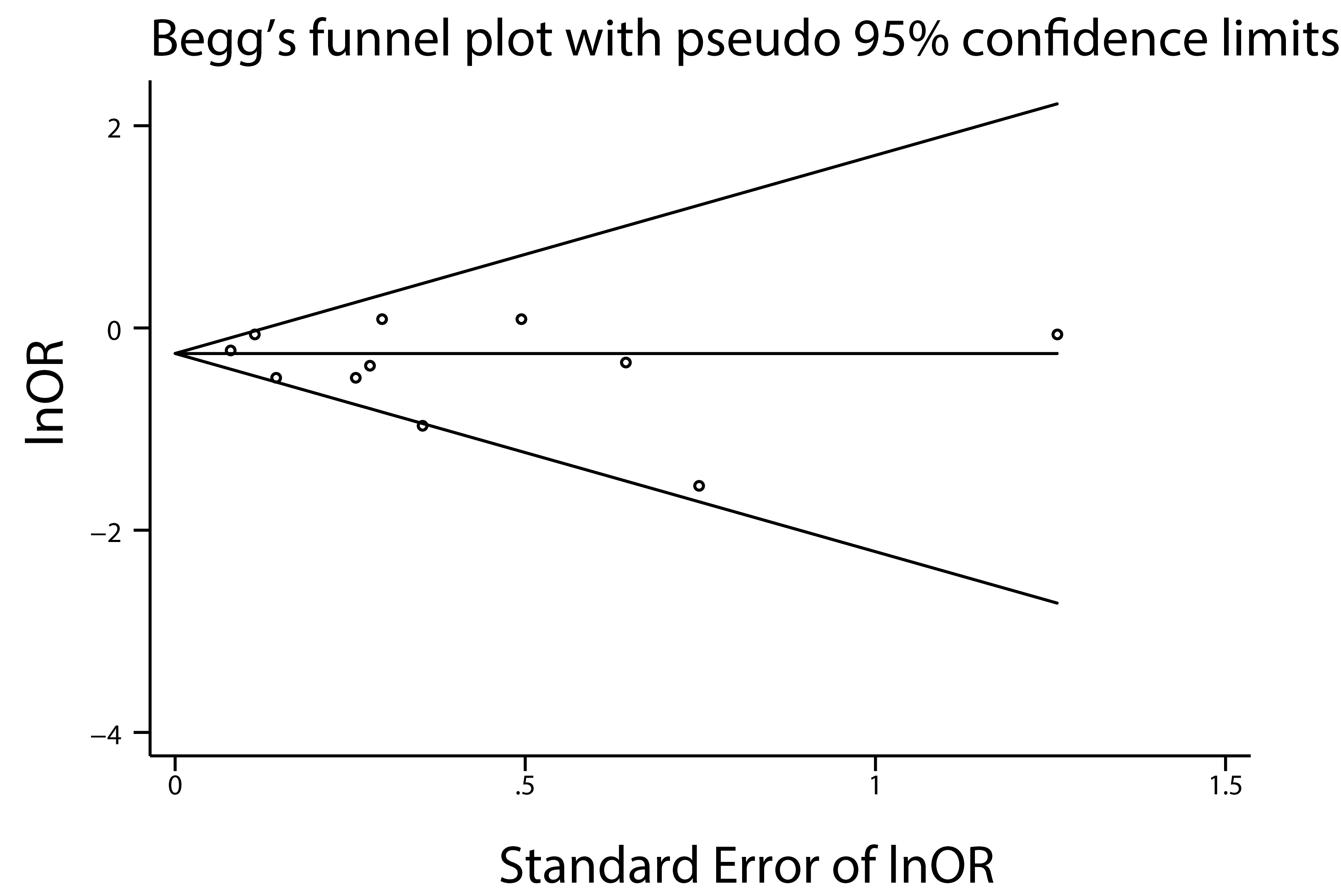

Supplement: Supplementary Figure 1 — Funnel plot assessing the mortality after preadmission metformin use in septic patients with DM. [file DataSheet_1.zip › Data sheet 1/Supplemental Figure 5.tif]

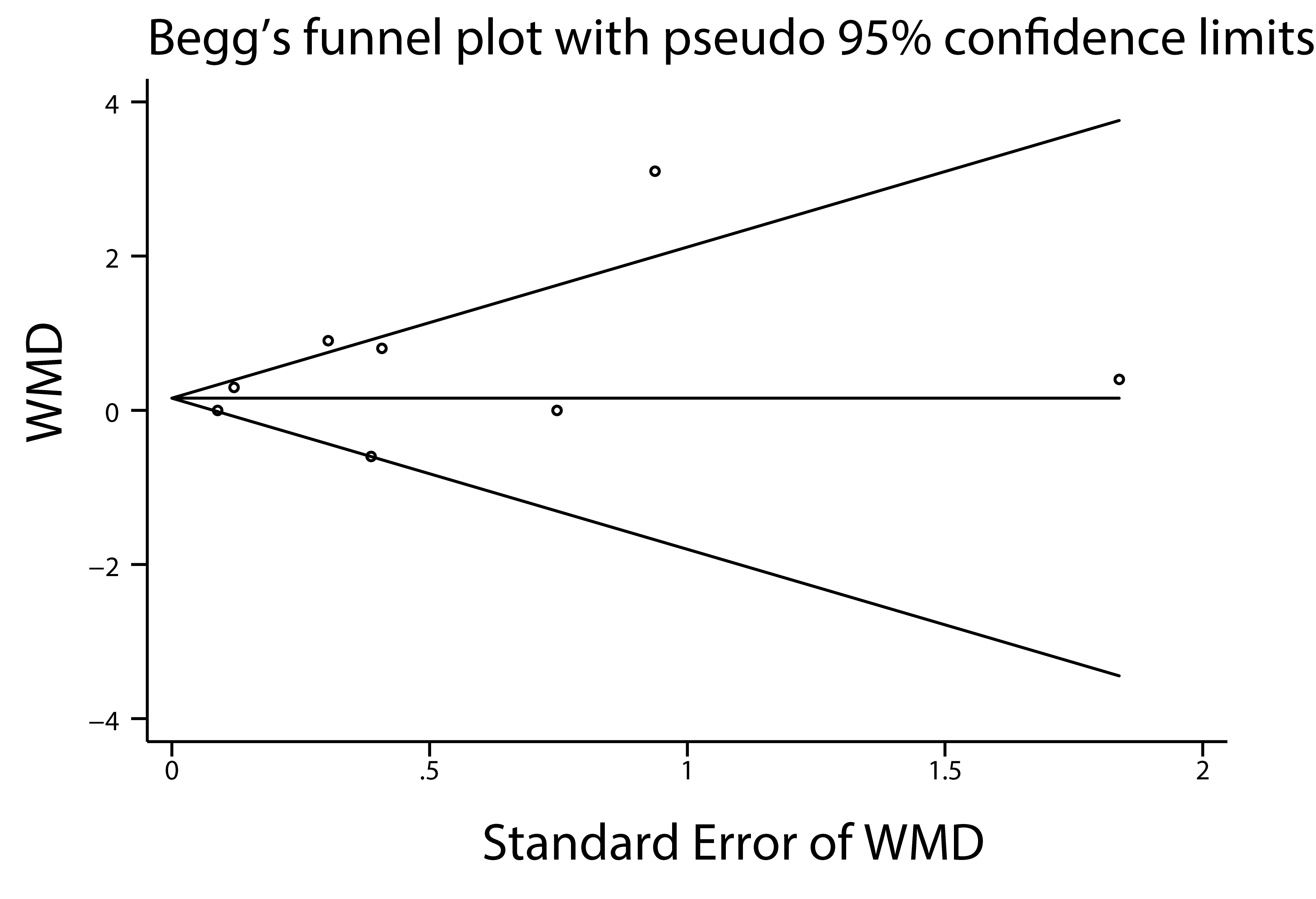

Supplement: Supplementary Figure 1 — Funnel plot assessing the mortality after preadmission metformin use in septic patients with DM. [file DataSheet_1.zip › Data sheet 1/Supplemental Figure 6.tif]
